# From Uptake to Therapeutic Function in Engineered Exosome Delivery Systems

**DOI:** 10.34133/research.1389

**Published:** 2026-08-12

**Authors:** Yiwei Li, Limin Jin, Pengli Gao, Mingmei Li, Zengkai Zhao, Jianjun Jiang, Ming An, Fangzhou Li

**Affiliations:** ^1^ Tianjin Key Laboratory of Biomedical Materials, Key Laboratory of Biomaterials and Nanotechnology for Cancer Immunotherapy, Institute of Biomedical Engineering, Chinese Academy of Medical Sciences and Peking Union Medical College, Tianjin 300192, PR China.; ^2^ College of Chemistry and Materials Science, Hebei University, Baoding, Hebei Province 071002, PR China.; ^3^ Bao Ding No. 1 Central Hospital, Baoding, Hebei Province 071002, PR China.

## Abstract

Engineered exosomes are increasingly recognized as promising vehicles for precision drug delivery. Genetic display, chemical modification, physical loading, and hybrid vesicle construction have expanded their applications, while introducing additional variables to the interpretation of delivery outcomes. Because engineering can alter vesicle properties, receptor engagement, endocytic routing, intracellular trafficking, and functional readouts, tissue accumulation or cellular uptake alone is insufficient to define effective delivery. This review traces engineered exosome delivery from cellular uptake to therapeutic function, covering engineering strategies, targeting validation, endocytic routing, intracellular fate, and functional readouts. We further discuss the strengths and limitations of commonly used evaluation methods. Across biomedical applications, we emphasize that delivery evidence should be judged according to cargo type, site of action, disease model, and therapeutic mechanism. By linking engineering design, assay selection, and evidence strength, this review supports a more careful interpretation of engineered exosome delivery.

## Introduction

When exosomes were first discovered in 1981, they were thought to be merely metabolic waste products of maturing red blood cells. However, as research on extracellular vesicles (EVs) has progressed, exosomes have been redefined as nanoscale membrane vesicles primarily formed via the endosomal pathway and released following fusion of multivesicular bodies with the cell membrane [1]. They are widely present in nearly all biological fluids and tissues [2,3], including blood, urine, saliva, and breast milk. As endogenous intercellular communication carriers, exosomes can transfer molecular substances, such as proteins, lipids, and nucleic acids, from donor cells to recipient cells [4]. Compared to synthetic nanocarriers, exosomes demonstrate unparalleled natural advantages in clinical drug delivery due to their higher biocompatibility and lower immunogenicity [5]. Exosomes also possess the innate ability to penetrate complex biological barriers such as the blood–brain barrier (BBB) [6] and can reduce tumor multidrug resistance by bypassing the P-glycoprotein efflux system [7]. Exosomes have become a promising platform for precision medicine in clinical practice [8].

To further enhance their targeting precision, loading efficiency, and intracellular delivery capacity, researchers have developed various engineered exosomes using advanced technologies such as genetic engineering of donor cells [9], in vitro chemical functionalization [10], physical drug loading [11], and biohybridization [12]. These engineered exosomes have shown excellent efficacy in various aspects such as tumor treatment, brain-targeted delivery, RNA and protein delivery, and immune regulation [13].

However, the biomedical performance of engineered exosomes should not simply depend on their accumulation or uptake in target cells but also be evaluated by whether they can achieve effective functional delivery and produce expected biological effect [14–17]. After internalization, cargos may remain trapped in endosomes, be degraded by lysosomes, recycle back to the plasma membrane, or reach functional intracellular sites. Without standardized qualitative and quantitative evaluation strategies, labeling artifacts, nonspecific cell association, and misunderstandings of endocytic pathways may lead to misjudgments of delivery efficiency and therapeutic potential.

**Fig. 1.**
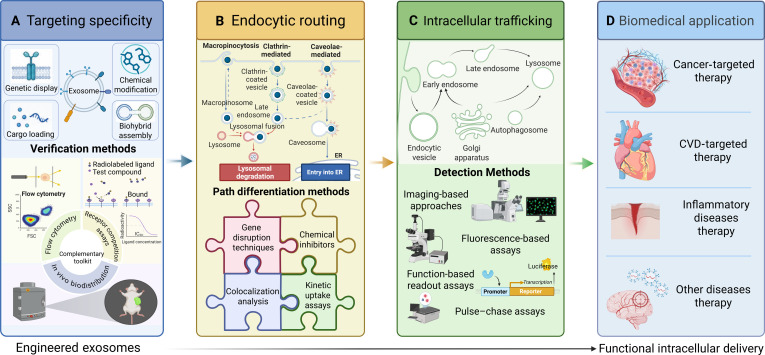
Overall evaluation framework. (A) Targeting specificity and verification of engineered exosomes. (B) Endocytic routing and pathway discrimination. (C) Intracellular trafficking and detection methods. (D) Biomedical application of engineered exosomes. IC_50_, half-maximal inhibitory concentration; SSC, side scatter; FSC, forward scatter.

This issue becomes more complex in engineered exosome systems, where surface display, chemical modification, physical cargo loading, and hybrid bioengineering may alter vesicle surface properties, receptor engagement, cellular association, intracellular trafficking, and functional readouts. Therefore, this review focuses on the distinction between apparent uptake and effective delivery to examine engineered exosome delivery as an integrated process. We summarize recent advances in exosome engineering, targeting specificity validation (Fig. 1A), endocytic-route analysis (Fig. 1B), intracellular-transport and endosomal-escape assessment (Fig. 1C), functional cargo release, and biomedical applications (Fig. 1D). Through comparing the strengths, limitations, and evidential value of different methods, this review provides a structured framework for evaluating engineered exosome delivery.

## Exosome Engineering Strategies and Targeting Specificity Validation Methods

To transform natural exosomes into precision therapeutic tools, researchers have developed diverse methodologies to confer cell type targeting specificity. Examples of engineered exosomes for targeted drug delivery are summarized in Table 1.

**Table 1.** Engineered exosomes for targeted drug delivery

Therapeutic cargo	Targeting ligand	Methods	Target cells	Function	Reference
BACE1 siRNA	RVG peptide	Lamp2b	Neuronal cells (Neuro-2a)	AD gene knockdown	[9]
GFP gene	CAP peptides	Lamp2b	CNS cells	Brain-wide expression	[21]
miRNA-26a	Apo-A1	CD63	HepG2 (liver)	Suppress proliferation	[29]
miRNA-let-7a	GE11 peptide	PDGFR	Breast cancer	Inhibit tumor growth	[33]
curcumin	c(RGDyK) peptide	Click chemistry	Endothelial cells (integrin αvβ3-positive)	Reduce inflammation and inhibit apoptosis in ischemic stroke	[10]
PTX	AA	DSPE-PEG-AA	Murine lung cancer (3LL-M27), sigma receptor-positive cells	Enhance drug tumor uptake and inhibit pulmonary metastases	[38]
siRNA	RNA aptamer, folate	cholesterol	Prostate/breast/colorectal cancer	Enhance tumor-specific targeting and siRNA efficient release	[39]
ESIONPs	-	Coincubation	Vascular endothelial cells	Suppress tumor angiogenesis and tumor growth	[11]
Corynoxine-B	Fe65 protein	Sonication	APP-expressing neurons	Mitigate AD cognitive decline	[46]
Triptolide/miR-497	RGD peptide	Membrane fusion	Ovarian cancer	Overcome cisplatin resistance	[47]
CRISPR/Cas9		Exosome–liposome fusion	MSCs	Gene editing efficiency	[12]

Lamp2b, lysosome-associated membrane glycoprotein 2b; RVG, rabies viral glycoprotein; siRNA, small interfering RNA; AD, Alzheimer’s disease; APP, amyloid precursor protein; BACE1, beta-site amyloid precursor protein cleaving enzyme 1; Apo-A1, apolipoprotein A1; PDGFR, platelet-derived growth factor receptor; DSPE-PEG, 1,2-distearoyl-sn-glycero-3-phosphoethanolamine-polyethylene glycol; AA, aminoethyl anisamide; PTX, paclitaxel; ESIONPs, extremely small iron oxide nanoparticles; MSCs, mesenchymal stem cells; CNS, central nervous system; GFP, green fluorescent protein; c(RGDyK), cyclic arginine-glycine-aspartic acid-D-tyrosine-lysine peptide; CAP, capsid-specific peptide

### Genetic surface-display strategies

**Fig. 2.**
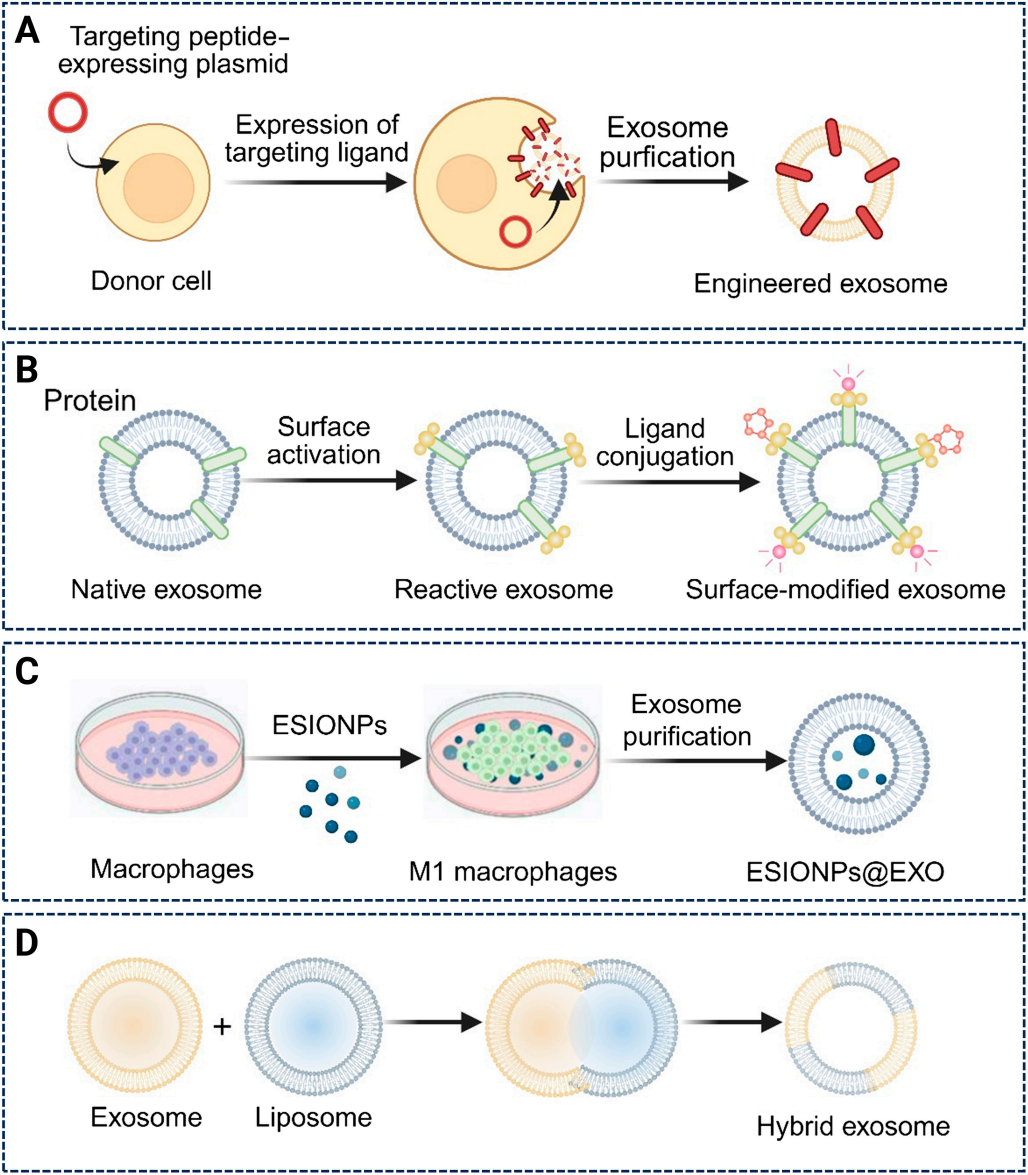
Exosome engineering strategies. (A) Lysosome-associated membrane glycoprotein 2b (Lamp2b)-mediated genetic surface-display. (B) Schematic illustration of 2-step chemical surface functionalization of exosomes. Adapted from [10] with permission. © 2017 Elsevier Ltd. All rights reserved. (C) Fabrication of ESIONPs@EXO by coincubating extremely small iron oxide nanoparticles (ESIONPs) with M1-polarized macrophages. Adapted from [11], published under CC BY 4.0. © 2024 The Authors. (D) Illustration of the procedure to produce hybrid exosomes.

Genetic surface-display strategies aim to present targeting ligands or functional proteins on engineered exosomes by leveraging endogenous membrane scaffolds that become incorporated into vesicles during biogenesis. It essentially fuses the gene sequence of a guiding protein or polypeptide with that of a selected exosomal membrane protein. A common approach utilizes exosome-enriched transmembrane proteins, such as lysosome-associated membrane glycoprotein 2b (Lamp2b), as fusion partners for peptides or protein domains of interest [18,19]. A landmark application of this strategy was reported in 2011, when Alvarez-Erviti et al. fused the neuron-specific rabies viral glycoprotein (RVG) peptide to the N-terminal transmembrane domain of Lamp2b, with the aim of delivering small interfering RNA (siRNA) across the BBB to address the therapeutic challenges of Alzheimer’s disease (AD) (Fig. 2A). The resulting engineered exosomes specifically targeted acetylcholine receptors on neurons, achieving a knockdown of beta-site amyloid precursor protein (APP) cleaving enzyme 1 (BACE1) messenger RNA (mRNA) and protein expression levels exceeding 60% in the mouse brain, without systemic toxicity observed [9,20]. More recently, Sarkar et al. [21] constructed chondrocyte-affinity peptide-exosomes by fusing various brain-targeting peptides with Lamp2b, thereby overcoming the problem of limited distribution in the brain associated with traditional carriers and achieving efficient gene delivery throughout the entire brain. This demonstrates that this strategy continues to be actively applied and continuously optimized to this day. However, the efficiency of Lamp2b-based display depends on fusion-protein design and endosomal processing during exosome biogenesis [22]. Donor-cell expression alone cannot verify that the ligand is retained on the surface of secreted exosomes [23]. Peptides fused to Lamp2b can be partially cleaved in the endosomal pathway reducing the amount of functional ligand retained on vesicles, but this instability can be mitigated by engineered glycosylation motifs. Larger ligands may impose additional constraints on protein folding, membrane insertion, or vesicle sorting, and these effects may vary among cell types [24]. However, immunoblotting of purified exosomes can confirm fusion-protein enrichment, whereas bead-assisted flow cytometry, immunogold electron microscopy, and protease-accessibility assays can further assess ligand stability and surface accessibility [25].

In addition to Lamp2b scaffolds, tetraspanins enriched on exosomes, including CD63, CD81, and CD9, have also been repurposed for surface display [26–28]. Fusion of targeting moieties or other functional domains to the transmembrane regions of these tetraspanins enables their presentation on the external face of the exosomal membrane, facilitating interactions with specific receptors on recipient cells.‍ For instance, Liang et al. [29] fused apolipoprotein A1 (Apo-A1) to the transmembrane region of CD63 to target scavenger receptor B1 overexpressing hepatocellular carcinoma cells, successfully suppressing tumor migration through the delivery of active miR-26a. The challenge of tetraspanin-based display is that CD63, CD81, and CD9 are functional components of EV membranes rather than neutral anchoring modules. Fusion of exogenous ligands may affect vesicle sorting, membrane organization, and endogenous cell-recognition properties [24,30]. Display efficiency is also constrained by scaffold topology. For example, full-length CD63 can limit terminal ligand exposure, leading to the development of engineered CD63-derived scaffolds with improved surface accessibility [31]. This scaffold dependence is also evident in antibody-displaying EVs, where the tetraspanin backbone influences both the incorporation of antibody-binding domains into EVs and their accessibility for target recognition [32]. Thus, enhanced uptake of tetraspanin-engineered exosomes should be interpreted together with scaffold-matched controls and receptor-blocking or receptor-negative models.

Moreover, the transmembrane protein platelet-derived growth factor receptor (PDGFR) is also frequently engineered for targeting delivery. By fusing the GE11m (YHWYGYTPQNVI) peptide to the transmembrane domain of PDGFR, exosomes can specifically target cancer cells that overexpress epidermal growth factor receptor [33]. However, receptor-derived scaffolds may retain trafficking or signaling motifs that influence donor-cell physiology, vesicle sorting, or endogenous cargo composition [24]. Therefore, PDGFR-based systems should be evaluated using receptor-defined models, receptor-blocking antibodies, or free-ligand competition assays, rather than relying on increased uptake as evidence of targeting specificity [23].

Overall, genetic surface display is a versatile strategy that exploits endogenous membrane proteins to achieve controlled presentation of targeting ligands on engineered exosomes. Selection of appropriate scaffolds and careful design of fusion constructs are essential considerations that influence both the extent of surface expression and downstream binding interactions with intended cell types.

The main limitation of genetic surface display is that successful donor-cell transfection does not necessarily mean uniform ligand presentation on exosomes. The fusion proteins may be degraded during vesicle formation [34], or remain in donor cells, or may be incorporated into only a subset of vesicles. Different scaffolds also introduce different risks. Lamp2b fusion peptides may require stabilization to avoid degradation, the tetraspanin-based display may alter natural tetraspanin interactions, and receptor-derived scaffolds such as PDGFR may change vesicle-cell signaling. Therefore, genetic surface display should verify both construct expression in donor cells and ligand presentation on isolated vesicles, preferably using vesicle-level assays such as immunoblotting, bead-based flow cytometry, nanoflow cytometry, or single-vesicle analysis. Functional evaluation should further include target-cell binding, receptor competition, and cargo-specific activity.

### Postisolation chemical modification

Although genetic engineering is highly effective for surface display of peptides or proteins, it is fundamentally constrained by the scope of genetically encodable targeting motifs. In contrast to genetic approaches, chemical modification is performed postisolation, offering a more rapid and modular methodology. This strategy enables a diverse array of natural and synthetic ligands to be displayed on the exosome surface through bioconjugation or lipid assembly. Among these, bio-orthogonal click chemistry has emerged as a powerful tool for site-specific modification. The amine groups of exosomal surface proteins can be easily functionalized with alkyne moieties, which then undergo copper-catalyzed azide-alkyne cycloaddition “click” reactions to bio-orthogonally couple to azide-containing reagents. For instance, in a study targeting ischemic stroke, researchers utilized a streamlined bio-orthogonal click reaction to conjugate the c(RGDyK) (cyclic arginine-glycine-aspartic acid-D-tyrosine-lysine peptide) (Arg-Gly-Asp-D-Tyr-Lys) peptide to the exosome surface [10] (Fig. 2B). These engineered exosomes exhibited superior BBB penetration and concentrated in lesion areas, delivering curcumin to markedly attenuate neuroinflammation and cellular apoptosis.

Metabolic glycoengineering offers another click-chemistry route that targets EV glycans rather than surface amines. Azido-sugar precursors, such as tetraacetylated N-azidoacetylmannosamine or tetraacetylated N-azidomannosamine, are incorporated into glycans in producer cells and passed to released EVs, generating azide-bearing vesicles for copper-free strain-promoted azide-alkyne cycloaddition with dibenzocyclooctyne- or cyclooctyne-modified probes, ligands, polymers, or immunostimulatory cargo. This strategy avoids copper catalysis and minimizes direct modification of native EV surface proteins. Bhatta et al. [35] used tetraacetylated N-azidomannosamine-labeled EVs and dibenzocyclooctyne-cargo conjugation to generate Toll-like receptor 9 agonist-modified EV vaccines, which enhanced dendritic-cell activation and antitumor T-cell responses. Related metabolic-labeling strategies have also been used to capture nascent tumor EVs in vivo during anti-programmed death-ligand 1 therapy [36] and to decorate EVs with PEGylated hyaluronic acid for CD44-mediated targeting [37]. Together, these studies extend click chemistry from amine coupling to glycan-directed EV surface editing for tracking, immune activation, and targeted delivery.

Another prevalent technique is hydrophobic insertion, which leverages the exosomal lipid bilayer to anchor amphiphilic molecules such as 1,2-distearoyl-sn-glycero-3-phosphoethanolamine–polyethylene glycol (DSPE-PEG). Kim et al. [38] utilized this approach to conjugate aminoethyl anisamide (AA) via a DSPE-PEG linker onto macrophage-derived exosomes to target sigma receptors overexpressed in lung cancer metastases. The DSPE-PEG-AA-modified exosomes showed enhanced internalization in lung cancer cell lines and improved the bioavailability and antitumor efficacy of paclitaxel (PTX) in pulmonary disease models. As DSPE-PEG is a Food and Drug Administration-approved module for clinical use, it has been widely adopted to facilitate the stable surface display of targeting motifs on exosomal membranes. Furthermore, cholesterol can self-assemble into the exosomal membranes due to its innate hydrophobicity, achieving effects comparable to DSPE. Pi et al. [39] utilized cholesterol anchors to conjugate AS1411 aptamers, targeting nucleolin on breast cancer cells to promote the delivery of let-7 microRNA and suppress tumor progression. Besides serving as structural membrane anchors, lipid species displayed on or incorporated in engineered exosomes may also participate in receptor-mediated signaling. For example, recent structural and functional studies identified formyl peptide receptor 2 (FPR2) as a receptor for long-chain ceramides and showed that C16:0, C18:0, and C20:0 ceramides can bind to the orthogonal pocket of FPR2, thereby regulating the G_i_ cyclic adenosine monophosphate signaling pathway signaling pathway [40]. This finding suggests that lipid modification on the exosome membrane may alter not only membrane composition and stability but also potential interactions with lipid-sensing receptors on recipient cells.

Nevertheless, the inherent complexity of the exosomal surface can lead to reduced efficiency in chemical modification reactions. Moreover, bioconjugation via click chemistry often lacks site-specific control, which may shield essential protein–protein interactions and compromise the structural integrity or biological function of the vehicle. Lipid self-assembly strategies have also been associated with potentially increased exosome toxicity. These limitations also affect how chemically modified exosomes should be evaluated. For click chemistry, ligand density, residual reagents, copper-related toxicity, and possible masking of natural surface proteins should be assessed [41]. As for lipid insertion, the stability of inserted ligands, changes in membrane fluidity, and altered interaction with lipid-sensing receptors should be considered. Recent structural work showing that long-chain ceramides can signal through FPR2 further suggests that lipid components may not be passive membrane anchors but rather receptor-active molecules [40]. Thus, lipid modification may influence both targeting behavior and recipient cell signaling and should be interpreted with appropriate controls. Regardless of these hurdles, chemical modification remains one of the most promising strategies for functionalizing the exosomal surface.

### Physical cargo loading and hybrid bioengineering approaches

Physical cargo loading methods are attractive because they can introduce small molecules, nucleic acids, proteins, or nanomaterials after vesicle isolation, but their effects on vesicle integrity and cargo activity vary greatly across methods. Current research has explored various physical approaches, including sonication, coincubation, and electroporation, to facilitate the encapsulation of targeted functional nanomaterials within the exosomal cavity rather than relying solely on surface modification. Coincubation mainly relies on passive diffusion, hydrophobic partitioning, or surface adsorption and is relatively mild, but its loading efficiency is often low, and free cargo contamination must be excluded [7,42]. Sonication can improve loading by temporarily disturbing the vesicle membrane, yet excessive sound energy may alter size distribution, membrane-protein conformation, or cargo stability [43,44]. Electroporation is frequently used for nucleic acids and CRISPR-associated cargos, but it may induce vesicle aggregation, RNA precipitation [45], membrane instability, or apparent loading artifacts. Therefore, loading efficiency should be interpreted together with characterization of loaded vesicles, removal of free cargo, and cargo-activity assays [23,42]. For example, Zhang et al. [11] constructed exosomes loaded with extremely small iron oxide nanoparticles (ESIONPs) (ESIONPs@EXO) by coincubating ESIONPs with classically activated (M1) macrophages (Fig. 2C). The study reported that ESIONPs@EXO inhibitedpathological angiogenesis both in vitro and in vivo, with no obvious toxicity. In 2023, Iyaswamy et al. [46] developed a highly innovative strategy for the treatment of AD. They engineered hippocampal neuron-derived exosomes to overexpress Fe65, a neuronal adapter protein that natively interacts with the APP. By employing sonication, the autophagy inducer corynoxine-B was loaded into these Fe65-engineered exosomes. This system effectively hijacked the APP signaling pathway, specifically inducing autophagy in APP-expressing neurons and ameliorating cognitive decline in AD mouse models.

Lipid bilayer membrane fusion is a key fundamental cellular process that has been successfully adapted for exosomal membrane engineering. During coincubation, liposomes harboring targeting motifs can spontaneously fuse with the exosomal membrane, allowing functional groups to be displayed on the surface of the resulting hybrid vesicles. For instance, Li et al. [47] developed a hybrid nanocarrier named hybrid exosome–liposome nanoparticles by fusing arginine–glycine–aspartic acid (RGD)-modified liposomes with CD47-bearing exosomes to specifically deliver triptolide and miR497 to tumor cells, thereby overcoming the prevalent chemoresistance in ovarian cancer. This hybrid architecture enables the system to evade clearance by the mononuclear phagocyte system while precisely homing to angiogenic tumor vessels, serving as a robust tool for cancer therapy. Additionally, hybrid exosomes fused with liposomes have been utilized for the targeted delivery of the CRISPR/Cas9 system into mesenchymal stem cells (MSCs), facilitating further advancements in in vivo genetic manipulation [12] (Fig. 2D).

Hybrid exosome–liposome systems also require careful interpretation. Fusion may improve loading capacity, but it can also generate mixed populations containing unfused exosomes, unfused liposomes, and hybrid vesicles [48]. As a result, uptake or functional changes may reflect altered lipid composition, residual liposomes, or cargo dose rather than a uniform improvement in exosome-mediated delivery. Fusion efficiency, free cargo removal, parental vesicle control, and cargo functional assays should be reported.

An issue shared by all engineering strategies is heterogeneity [23,48]. Engineered preparations may contain vesicle subpopulations with different ligand densities, membrane compositions, and biological activities. Genetic overexpression, incomplete chemical conjugation, variable physical loading, or incomplete exosome–liposome fusion can all amplify this heterogeneity. Therefore, average particle concentration or overall fluorescence is insufficient to define the engineered product. When conditions permit, engineered exosomes should be evaluated using particle-normalized and cargo-normalized dosing, batch comparison, ligand-density measurement, and single-particle or subpopulation-resolved analysis [23].

### Methods to validate targeting specificity

In exosome delivery studies, targeting specificity should be evaluated in 3 linked but distinct layers, namely, target recognition, cellular internalization, and functional cargo activity. Target recognition includes ligand–receptor binding or preferential tissue association. Cellular internalization refers to the entry of vesicles into intracellular compartments. Functional cargo activity requires a measurable biological effect caused by the delivered cargo. Therefore, validating targeting specificity demands assays that can differentiate receptor-mediated binding or uptake from nonspecific association and functional cargo delivery.

Among the most widely used in vitro techniques is flow cytometry [49,50], which enables quantitative analysis of exosome binding and internalization at the single-cell level [51]. Fluorescently labeled exosomes can be incubated with target and control cell populations, and the resulting fluorescence intensity profiles reveal differences in cellular association that reflect the contribution of engineered targeting ligands versus baseline nonspecific association. The inclusion of appropriate negative controls is essential to exclude nonspecific signals, such as comparative conditions performed with unlabeled exosomes or in the presence of blocking antibodies to assess background binding and uptake [52,53]. Quantitative normalization is also essential in cellular uptake assays. Signals should be normalized to particle number, cargo amount, labeling efficiency, and cell number. Otherwise, uncorrected differences in vesicle dose or fluorescence intensity can lead to overestimation of targeting or internalization efficiency [50]. For example, Zhao et al. [49] developed anti-CD19-engineered exosomes loaded with methotrexate (MTX) for central nervous system (CNS) lymphoma and used flow cytometry to assess their in vitro targeting behavior. Anti-CD19-Exo-MTX showed preferential association with CD19-positive SU-DHL-8 lymphoma cells, while only limited association was observed in CD19-negative 293T cells, supporting the contribution of CD19-mediated recognition to the engineered delivery system. Similarly, Wiklander et al. [32] generated antibody-displaying Fc-EVs and used flow cytometry-based analysis to compare EV uptake in antigen-positive and antigen-negative tumor cells. Human epidermal growth factor receptor 2-guided or programmed death-ligand 1-guided Fc-EVs showed increased uptake in the corresponding antigen-positive cells, providing evidence that antibody display can improve receptor-associated cell targeting. These examples show that flow cytometry is particularly useful when ligand-engineered vesicles are evaluated together with receptor-negative cells, nonengineered or isotype-control vesicles, and quantitative normalization. The interpretation can be further strengthened by combining flow cytometry with surface-fluorescence quenching, acid washing or protease stripping, confocal z-stack imaging, receptor-blocking assays, and cargo-specific functional readouts, which together help distinguish receptor-associated binding, internalization, and downstream cargo activity [54,55].

Receptor competition assays offer a complementary approach to confirm specificity by directly interrogating the interaction between targeting moieties and their cognate receptors. In such assays, preincubation of target cells with excess free ligand or receptor-blocking antibodies should reduce subsequent uptake of engineered exosomes if the interaction is truly receptor-dependent [56,57]. These competition experiments help discriminate between ligand-specific binding and generalized endocytic uptake pathways and are particularly useful when working with peptide- or antibody-based targeting motifs. This is particularly important for RVG, RGD, GE11, Apo-A1, or antibody-modified exosomes. If the uptake decreases after free-ligand competition or receptor block, it can provide stronger evidence that uptake depends on the intended receptor rather than on nonspecific adsorption or high vesicle dose.

While in vitro assays are informative, definitive evidence of targeted delivery often requires validation in whole organisms. In vivo biodistribution studies involve systemic administration of exosomes labeled with tracers (e.g., fluorescent [58], bioluminescent [59], or radiolabeled reporters [60]) followed by quantification of their distribution across organs and tissues over time [28,59]. For example, differential biodistribution patterns observed between targeted and untargeted exosome preparations in rodent models provide mechanistic insight into how surface engineering alters organ tropism, clearance, and target engagement. In this context, appropriate controls are essential for interpreting in vivo biodistribution, particularly because the detected signals report labeling tracers rather than the exosomes themselves. Animals treated with equivalently prepared but nontargeted exosomes are recognized as control, allowing differences to be attributed to targeting modifications. In parallel, control groups treated with free tracer are necessary to assess background signals from unbound probes.

In addition to conventional flow cytometry- and imaging-based assays, emerging dual-mode biosensing platforms may further improve the reliability of exosome detection and surface-marker validation. For example, Lu et al. [61] developed a peptide–graphene sensing system using the CD63-binding peptide CP05 and graphene oxide for dual-signal detection of tumor-derived exosomes through fluorescence and resonance light scattering responses. This approach enables quantitative detection of CD63 and tumor-derived exosomes and provides orthogonal signal readouts that may reduce dependence on a single labeling modality. However, such platforms primarily support exosome identification and marker-associated detection. Thus, they should be integrated with uptake assays, receptor-blocking experiments, biodistribution analysis, and functional cargo readouts before being used to support claims of targeting specificity or functional delivery.

Collectively, flow cytometry, receptor competition, dual-mode detection, and in vivo biodistribution imaging provide complementary evidence [28,59]. Flow cytometry mainly quantifies cellular association, competition assays test receptor dependence, dual-mode sensing improves marker-level detection, and biodistribution imaging evaluates target site exposure. None of these methods alone proves functional delivery [24]. Their value lies in being combined with intracellular trafficking and cargo-specific functional assays in later sections.

## Endocytic Routing and Pathway Discrimination

### Main endocytic routes

**Fig. 3.**
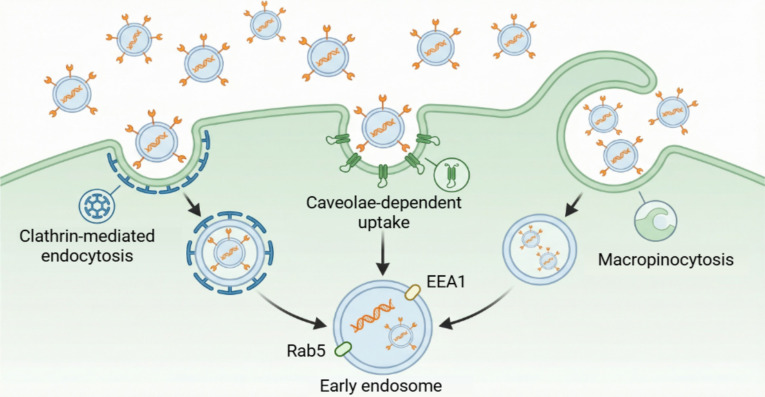
Endocytic pathways and distinguishing methods comparison. Exosomes enter cells via clathrin-mediated endocytosis, caveolin-dependent endocytosis, or macropinocytosis, converging in early endosomes. These distinct entry pathways influence intracellular transport and subsequent fate to varying degrees.

The cellular uptake of exosomes occurs through a repertoire of endocytic pathways [62,63], with clathrin-mediated endocytosis [64], caveolin-dependent uptake [65], and macropinocytosis [66] representing 3 principal mechanisms implicated in a range of cell types (Fig. 3). It is noteworthy that these pathways often coexist, with the specific dominant pathway determined by the vesicle subtype, surface characteristics, and the type and physiological state of the recipient cell [63].

Clathrin-mediated endocytosis is a well-characterized, receptor-dependent process, which is classically initiated by ligand–receptor engagement at the cell surface. However, emerging evidence indicates that paracrine adhesion signaling from the extracellular matrix, such as the clustering of integrin β1 and talin-1, can also trigger this pathway by activating receptor tyrosine kinases and downstream calcium signals [65]. This cascade leads to the formation of clathrin-coated pits that invaginate and pinch off from the plasma membrane. Following internalization, clathrin coats are shed and cargo is delivered to early endosomes. This compartment acts as a key sorting station that directs cargo either toward recycling pathways or toward late endosomes and lysosomes, depending on molecular features of the internalized cargo and the cellular context [67,68].

Caveolin-dependent endocytosis is a specialized pathway that utilizes flask-shaped plasma membrane invaginations known as caveolae. These microdomains are enriched in cholesterol, caveolin proteins like Caveolin-1 (CAV1), and associated cavin proteins, which are essential for their formation and function [69]. Certain populations of exosomes exploit this route for cellular entry. Notably, the contribution of caveolar uptake can vary substantially depending on the recipient cell type and the specific surface protein context of both the exosomes and the target cell [67].

Lipid-dependent signaling may further complicate the interpretation of receptor-mediated exosome uptake. Ceramides are bioactive sphingolipids involved in membrane organization, vesicle formation, and stress signaling. Recent work identified FPR2 as a receptor for long-chain ceramides and showed that C16:0, C18:0, and C20:0 ceramide can activate FPR2-dependent G_i_ cyclic adenosine monophosphate signaling pathway signaling in adipocytes [40]. Although direct evidence connecting ceramide-FPR2 signaling to engineered exosome internalization is still lacking, this finding suggests that lipid composition and lipid-sensing receptors should be considered when interpreting exosome uptake pathways.

Macropinocytosis is a distinct and actin-driven endocytic process characterized by the formation of large membrane ruffles that fold back onto the cell surface and engulf extracellular fluid and particles without apparent receptor specificity [70]. This pathway generates large vesicles called macropinosomes, which subsequently merge with endosomal compartments. In the context of exosomes uptake, macropinocytosis is notable for its capacity to internalize vesicles across a broad-size spectrum, making it a potentially efficient route for larger microvesicles [66]. Its involvement is frequently identified using pharmacological inhibitors and confirmed via live-cell imaging.

In addition to ligand–receptor recognition on the vesicle surface, the extracellular microenvironment may also shape receptor-mediated uptake by altering receptor conformation and downstream signaling. Acidic pH is a common feature of tumors, ischemic tissues, and inflamed microenvironments, all of which are major target sites for engineered exosome therapy. Recent structural work on the proton-sensing G protein-coupled receptor GPR4 showed how extracellular protonation can induce receptor activation and species-specific pH sensitivity, providing a mechanistic example of how pH-dependent receptor states may influence cellular signaling under pathological conditions [71]. Although GPR4 has not been established as a direct exosome uptake receptor, this study highlights that receptor-mediated exosome internalization should be interpreted in the context of local pH, receptor activation state, and recipient-cell physiology.

Critically, these multiple endocytic pathways often coexist within the same cell system. This heterogeneity implies that engineering strategies designed to enhance overall exosomes uptake, such as modifying surface charge or attaching specific ligands, may not uniformly bias vesicles toward a single predetermined intracellular route [67]. Instead, the engineered surface features are likely to differentially modulate the engagement of clathrin-mediated, caveolar, and macropinocytic pathways in a manner that is highly dependent on the target cell’s biological context. This underscores the importance of employing systematic, pathway-aware evaluation approaches when developing and characterizing engineered exosomes delivery platforms.

### Experimental approaches to distinguish pathways

Dissecting the specific endocytic pathways involved in exosome uptake often requires a combination of pharmacological, genetic, and imaging-based strategies [72] (Table 2).

**Table 2.** Orthogonal strategies for evaluating endocytic pathway involvement and their main limitations

Pathway	Chemical inhibitors	Main limitation	Genetic validation	Imaging / kinetic validation	Preferred evidence combination	Applicable scenarios
Clathrin-mediated	Dynasore	Inhibits dynamin-dependent vesicle scission but may also affect cholesterol homeostasis and lipid raft organization.	CLTC, AP2, and DNM2 knockdown/knockout. Rescue if possible.	Clathrin/ AP2 colocalization; kinetic single-particle tracking	Inhibitor response + CLTC/AP2/DNM2 perturbation + early-entry imaging + receptor-blocking assay	Ligand-engineered exosomes; receptor-defined tumor or epithelial cells
Caveolin/ lipid raft-associated	MβCD	Depletes membrane cholesterol and broadly disrupts cholesterol-rich membrane domains, including lipid rafts and caveolae.	CAV1 knockdown/knockout. Cholesterol rescue after MβCD.	Caveolin-1 colocalization; membrane domain imaging	Cholesterol perturbation + CAV1 perturbation + cholesterol rescue + barrier controls	Endothelial cells, BBB models, lipid raft-rich membranes, caveolae-positive recipient cells
Macropinocytosis	EIPA	Inhibits Na^+^/H^+^ exchange and may alter actin remodeling. Its effect may vary with cell metabolism and membrane ruffling activity.	RAC1, PAK1, CDC42, PI3K pathway perturbation.	High-molecular-weight dextran uptake; membrane ruffling; live-cell imaging	EIPA + dextran uptake + membrane-ruffling imaging + RAC1/PAK1/CDC42 perturbation.	Macrophages, dendritic cells, microglia, and tumor cells with strong fluid-phase uptake
Membrane fusion or nonendocytic entry	Low-temperature inhibition; fusion inhibitors when available	Dye transfer, close membrane apposition, or lipid mixing may mimic fusion. Conventional endocytosis inhibitors may not fully block this route.	Perturbation of candidate fusion proteins if known.	Content-mixing assay; split-reporter fusion assay; cytosolic cargo-accessibility assay	Mixed-signal detector + cooling control + functional cargo readout	Low-pinocytic brain endothelial cells, neurons, and fusion engineered exosomes

BBB, blood–brain barrier; CAV1, Caveolin-1; MβCD, methyl-β-cyclodextrin; EIPA, 5-(N-ethyl-N-isopropyl)amiloride; CLTC, clathrin heavy chain; DNM2, Dynamin-2; AP2, adaptor protein 2; CDC42, cell division control protein 42 homolog; PAK1, p21-activated kinase 1; RAC1, Ras-related C3 botulinum toxin substrate 1; P13K, phosphoinositide 3-kinase

**Fig. 4.**
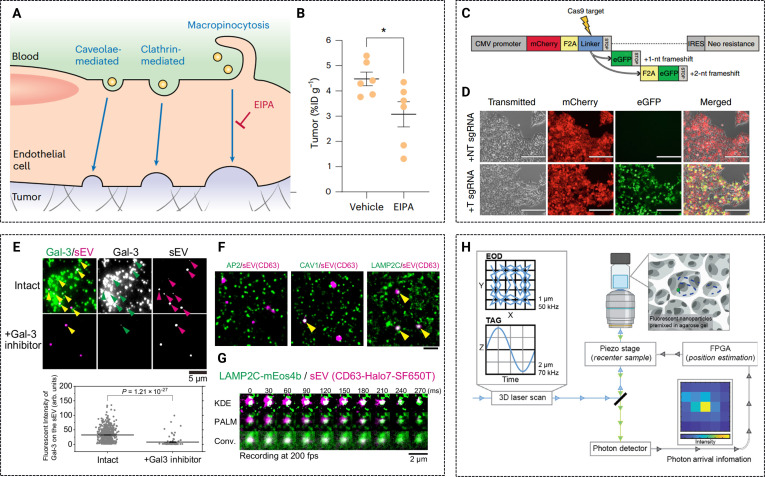
Experimental strategies for dissecting endocytic pathways involved in exosome uptake. (A) Pharmacological inhibition is used to block endocytic pathways and assess their contribution to nanoparticle or exosome uptake. (B) Decreased internalization after inhibitor treatment suggests possible pathway involvement. Reproduced from [76] with permission. © 2025 The Authors. (C and D) Schematics showing the CRISPR/Cas9-activated fluorescent stoplight reporter system. Reproduced from [78] under CC BY 4.0. © 2020 The Authors. (E to G) Live-cell and single-particle imaging of vesicle internalization. Reproduced from [65] under CC BY 4.0. © 2025 The Authors. (H) Single-particle tracking and kinetic analysis. Reproduced from [89] with permission. © 2025 Wiley-VCH GmbH.

One widely used experimental approach employs chemical inhibitors that selectively interfere with components of defined uptake mechanisms. However, these inhibitors are not strictly specific to their intended pathways. For instance, dynasore suppresses dynamin guanosine triphosphate (GTPase) activity and inhibits clathrin-dependent endocytosis by blocking dynamin-mediated vesicle scission [73]. Dynasore can also influence cholesterol homeostasis, lipid raft organization, and other dynamin-independent cellular processes [74]. Similarly, agents like methyl-β-cyclodextrin (MβCD) disrupt cholesterol-rich membrane domains and inhibit caveolin-associated uptake [75]. However, this treatment broadly perturbs plasma membrane organization, receptor clustering, membrane fluidity, and multiple cholesterol-dependent signaling domains. The macropinocytosis inhibitor (5-(N-ethyl-N-isopropyl)amiloride [EIPA]) is often used as a macropinocytosis inhibitor [76] (Fig. 4A and B), yet its inhibition of Na^+^/H^+^ exchange can alter intracellular pH, actin remodeling, and cell viability. Thus, inhibitor-sensitive uptake remains useful as supportive evidence for pathway involvement but should not be used alone for definitive pathway assignment, especially for inhibitors with known off-target effects such as dynasore, MβCD, or EIPA. These results should be interpreted together with orthogonal evidence [77].

Genetic perturbation techniques, including siRNA-mediated knockdown and CRISPR/Cas9-based gene editing, offer an orthogonal and often more specific avenue to interrogate endocytic pathway involvement [72,78] (Fig. 4C and D). By reducing the expression of core structural proteins involved in clathrin-mediated endocytosis, including components of the clathrin coat like the clathrin heavy chain (CLTC) [79], Dynamin-2 (DNM2) or key adaptor proteins such as AP2 (adaptor protein 2) [80], researchers can directly assess their contribution to exosome internalization relative to nontargeting controls [81]. Similarly, silencing CAV1 expression can elucidate the role of caveolar endocytosis [82]. While genetic methods generally provide superior molecular specificity compared to pharmacological inhibition, they require rigorous validation of knockdown/knockout efficiency and controls for compensatory cellular adaptations. Genetic perturbation should also be interpreted with caution. Knockdown of CLTC, DNM2, CAV1, or p21-activated kinase 1 may change membrane organization, receptor recycling, cytoskeletal state, or cell fitness, thereby indirectly affecting exosome uptake. Therefore, genetic inhibition is most informative when accompanied by rescue experiments, cell-viability controls, receptor-expression analysis, and confirmation that the same manipulation affects a canonical pathway marker in the expected direction.

In addition to perturbation approaches, high-resolution microscopy-based colocalization analysis provides direct visual evidence for the involvement of specific endocytic routes [79]. By fluorescently labeling exosomes and costaining for pathway-specific markers, such as clathrin for clathrin-mediated endocytosis or CAV1 for caveolae-dependent uptake, researchers can assess whether internalized vesicles are spatially associated with the expected uptake machinery. Recent live-cell and single-particle imaging studies further extend this approach by mapping vesicle uptake in relation to surface adhesion molecules and endocytic membrane domains, helping to interpret internalization events in their spatial and temporal context [65,83–85] (Fig. 4E to G). These imaging strategies provide spatial context and, when combined with time-course experiments, contribute valuable insights into the temporal sequence of internalization events [86]. However, static colocalization with clathrin, CAV1, or dextran-positive compartments does not by itself establish the route of entry, because vesicles may encounter these markers after internalization or during downstream sorting. For pathway assignment, colocalization should be time-resolved and ideally captured during the early entry window.

Lastly, to transition from static snapshots to dynamic quantification, single-particle tracking and kinetic uptake assays are indispensable. By monitoring the dynamics of individual fluorescently labeled exosomes in live cells over extended periods, it quantitatively resolves the kinetic mechanisms of exosome internalization and transport, revealing detailed kinetic parameters such as rates of initial binding, entry, and early intracellular movement [87,88]. This enables researchers to distinguish rapid uptake from slower nonspecific processes. Moreover, single-particle tracking can identify potential transport bottlenecks, such as prolonged retention within specific cellular compartments. Cutting-edge single-particle tracking techniques capable of high-speed 3-dimensional tracking with nanometer precision prove particularly powerful for such investigations [89] (Fig. 4H). Nevertheless, kinetic separation does not automatically identify molecular mechanism. Similar entry speeds may arise from different pathways, and the same pathway may show different kinetics across cell types. Therefore, single-particle tracking is best used to refine pathway hypotheses proposed by perturbation and marker-imaging experiments.

The priority of method combinations should be adjusted according to recipient-cell type. In endothelial or BBB-related models, where caveolae, lipid rafts, and transcytosis-related processes may be prominent, MβCD-based experiments should be paired with CAV1 perturbation, cholesterol rescue, and endothelial barrier-integrity controls. In macrophages, monocytes, dendritic cells, or microglia, macropinocytosis are often strong background routes; therefore, EIPA or actin inhibitors should be combined with dextran uptake, membrane-ruffling imaging, and immune-cell activation controls. In epithelial and many tumor cells, receptor-mediated clathrin-dependent uptake may be more relevant for ligand-engineered exosomes, but dynasore-sensitive uptake should still be validated with CLTC/AP2/DNM2 perturbation and receptor-blocking assays. In neurons or low-pinocytic brain endothelial cells, reduced uptake after conventional endocytosis inhibition should not exclude alternative entry routes such as membrane fusion, which requires content mixing or split-reporter assays.

In summary, a robust experimental framework for dissecting the complex endocytic pathways engaged during exosome uptake requires a multimodal approach [90]. The strategic combination of pharmacological inhibition, genetic manipulation, quantitative colocalization imaging, and kinetic single-particle tracking offers complementary lines of evidence, enabling a more comprehensive and conclusive understanding of the cellular entry mechanisms.

## Intracellular Trafficking of Engineered Exosomes

Assessing the intracellular fate of engineered exosomes should be regarded as a sequential process comprising a series of mechanisms and methodological challenges rather than a singular end point. This journey begins with compartmentalization following endocytosis, with the transition from early to late endosomes represents a critical juncture. This transition is typically traced through Rab5-to-Rab7 conversion [91] and markers such as early endosome antigen 1 (EEA1) [92] and lysosome-associated membrane protein 1 (LAMP1) [93], laying the foundation for different fates including degradation, recycling, or potential cytoplasmic release. Thus, clarifying compartmentalization dictates which detection methods hold practical relevance.

Moreover, to reliably demonstrate endosomal escape, colocalization analysis alone is insufficient. It should be complemented by pH-sensitive probes [94], galectin recruitment assays [95], and crucially, functional readouts (such as reporter gene expression, enzyme activity rescue, or cleavage reporter complementation) providing multiple lines of evidence for cytoplasmic accessibility. Meanwhile, a growing number of research indicates that Golgi-associated retrograde transport and trafficking pathways may also divert cargo from degradation fate [96]. Consequently, any analysis that neglect outward recycling risks yielding incomplete conclusions.

**Fig. 5.**
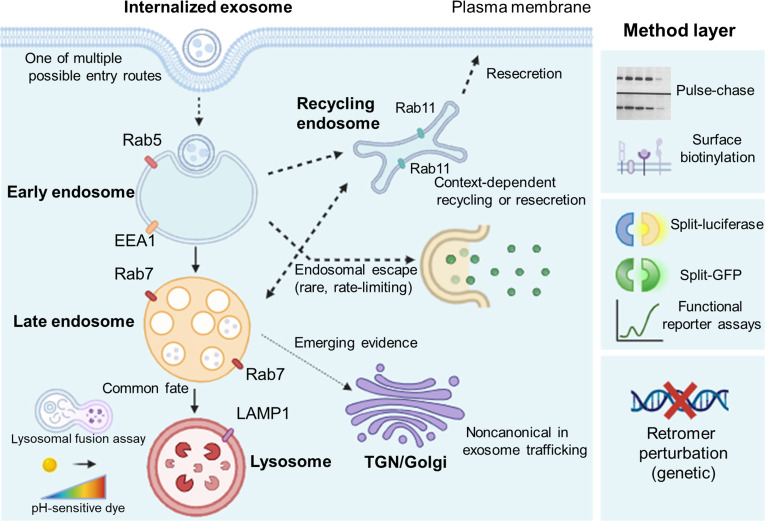
Context-dependent intracellular trafficking routes of internalized exosomes and representative methodological interrogation strategies. It highlights the process of the early endosome transforming into the late endosome, with its branching outcomes encompassing numerous pathways. The method layer on the right summarizes the representative detection strategies used to depict these pathways.

Methodological rigor is paramount throughout the research process. Quantitative kinetic measurements, the utilization of multiple complementary techniques, and meticulously designed positive and negative controls are essential to avoid experimental artefacts introduced by labeling, inhibitors, or overexpression. Therefore, trafficking analysis should combine quantitative colocalization, kinetic measurements, and perturbation experiments rather than relying on end point fluorescence overlap alone (Fig. 5).

### Endosomal maturation and intracellular sorting

#### Markers and probes for endosomal stage identification

Identification of endosomal stages during intracellular trafficking is commonly inferred from the use of molecular markers and probes that are enriched in specific endosomal compartments. Early endosomes are primarily defined by the presence of the small GTPase Rab5 and its key effector EEA1 [92]. EEA1 binds to Rab5-guanosine triphosphate in a phosphatidylinositol 3-phosphate-dependent manner, an interaction critical for tethering and fusion events at the early endosomal membrane [92,97]. These Rab5-positive compartments exhibit a mildly acidic lumen and function as the principal sorting station for internalized cargo, determining its subsequent itinerary within the endosomal network.

A pivotal event in endosome maturation is the regulated exchange of GTPase identity, termed “Rab conversion” [91]. In this process, Rab5 is progressively replaced by Rab7, a marker of late endosomes and multivesicular bodies. Rab7-enriched compartments are more acidic and engage machinery directing vesicles either toward lysosomal degradation or alternative fates. This Rab5-to-Rab7 switch is thus a conserved and widely utilized indicator of endosomal maturation across diverse biological systems [91].

At later stages of the endosomal–lysosomal continuum, lysosomal markers such as LAMP1 become increasingly enriched [93,98,99]. LAMP1 and related glycoproteins are abundant on lysosomal membranes and can be visualized using antibodies or fluorescent fusion constructs to delineate degradation-competent organelles. Respectively, Rab5 and EEA1 are commonly used to distinguish early endosomes, whereas Rab7 and LAMP1 indicate progression to late endosomes and lysosomal compartments [91,100]. The combined use of these markers often in conjunction with pH-sensitive probes or fluorescent tracers, allows researchers to map the progression of endosomal maturation and infer the intracellular routing of internalized vesicles.

#### Imaging-based approaches to track endosomal progression

Imaging-based approaches are indispensable for directly monitoring the endosomal progression of internalized vesicles, providing spatial and temporal insights that cannot be obtained from biochemical assays alone. Confocal microscopy remains one of the most widely used modalities for visualizing subcellular localization due to its optical sectioning capability and high spatial resolution. Confocal microscopy provides optical sectioning that minimizes out-of-focus light, facilitating spatial resolution of overlapping compartments. By performing 3-dimensional visualization of fluorescently labeled compartments in fixed cells, confocal imaging provides snapshots of vesicle progression from early to late stages in the endocytic pathway [101].

To move beyond static snapshots, live-cell imaging is essential for capturing the dynamic behavior of endosomal compartments in real time [102]. By employing fluorescent fusion proteins or organelle-specific dyes, researchers can track the movement, maturation, and interactions of endosomes over time. This approach is particularly powerful for revealing transient events and kinetic parameters, such as the rate of vesicle movement or the duration of organelle contacts, that are invisible in end point assays. Recent advances in multiplexed live-cell imaging, achieved through the integration of spinning-disk confocal microscopy with deep learning-based analysis, now permit the simultaneous tracking of multiple organelles [102]. This capability represents a substantial enhancement in dissecting complex trafficking networks.

Building on imaging data, colocalization analysis provides a quantitative measure of the spatial association between internalized vesicles (e.g., labeled exosomes) and specific endosomal compartment markers (e.g., Rab5, Rab7, and LAMP1) [101]. Coefficients such as Pearson’s correlation or Manders’ overlap are commonly calculated from multichannel confocal images to objectively assess the degree of overlap, helping to chart the cargo’s journey through the endocytic network.

In summary, the strategic integration of confocal microscopy, live-cell dynamic imaging, and quantitative colocalization analysis constructs a multidimensional picture of intracellular trafficking. This integrated imaging framework is critical for elucidating how engineered exosomes navigate from initial uptake sites through various endosomal and lysosomal compartments.

### Endosomal escape

For engineered exosomes, cellular internalization is only the initial step toward effective delivery. A decisive determinant of downstream activity is postuptake processing, particularly endosomal escape, which enables vesicle-associated cargo to access the cytosol through disruption or remodeling of the endosomal membrane [103]. Without efficient escape, internalized exosomes are predominantly trafficked toward lysosomal compartments, leading to cargo degradation and loss of bioactivity [104]. This bottleneck is highlighted by studies on synthetic nanoparticles, which estimated that only about 1% to 2% of internalized particles successfully reach the cytoplasm [105]. These observations highlight endosomal escape as a fundamental bottleneck rather than a secondary consideration. Consequently, rigorous evaluation of endosomal escape efficiency is essential for determining whether an engineered exosome system can achieve efficient intracellular delivery. The following section will detail the methodologies designed to quantify this crucial event.

#### Fluorescence-based assays and common false positives

Fluorescence-based assays are widely employed to assess the extent to which internalized vesicles escape from endosomal compartments into the cytosol. One commonly used strategy involves the use of pH-sensitive fluorescent probes [94] that exhibit quenched emission under acidic endosomal conditions but increase in fluorescence upon exposure to the relatively neutral cytosolic environment, thereby providing an indirect readout of endosomal membrane disruption and cargo release. However, pH-sensitive fluorescence should be interpreted cautiously. Recent single-endosome analyses of RNA nanodelivery showed that endosomal membrane damage and detectable cargo release are not always simultaneous. Only a subset of damaged endosomes contained detectable RNA cargo, and only part of the cargo signal was rapidly released after membrane damage [106]. Thus, pH-responsive or dye-based signals are useful for identifying potential escape events, but their interpretation is stronger when linked to cargo tracking or downstream activity. Such pH-responsive indicators have been applied in studies of engineered delivery systems to detect changes in local pH as vesicles transition from endosomes to the cytoplasm [107]. Joshi et al. [108] used correlative light and electron microscopy for detecting endosomal lysis and cytosolic cargo exposure after EV uptake. By pairing fluorescent green fluorescent protein-tagged CD63 EVs with a cytosolic anti-green fluorescent protein nanobody reporter in recipient cells, they used fluorescence to detect cargo access to the cytosol and correlated these signals with ultrastructural evidence of endosomal escape.

A complementary membrane-damage assay relies on the recruitment of galectins to compromised endosomal membranes. Galectins are soluble cytosolic proteins that bind to β-galactoside sugars normally sequestered within the endosomal lumen. When endosomal membranes rupture, these sugars become exposed to the cytosol, triggering rapid recruitment of fluorescently tagged galectins [95]. Galectin-based sensors such as Galectin-9 have been shown to provide a robust and quantitative indicator of endosomal escape events in high-throughput imaging assays, with reporter accumulation marking sites of membrane perturbation [109]. Nevertheless, galectin recruitment reports exposure of luminal glycans after endosomal membrane damage, not direct cargo-release readout. It may also vary across cell types and can be influenced by membrane repair responses or inflammatory signaling triggered by endosomal damage. Thus, galectin assays should be combined with cargo-specific activity readouts.

Imaging-based methods become more informative when combined with cytosolic-accessibility reporters. The split luciferase endosomal escape quantification (SLEEQ) assay provides quantitative information on whether a delivered molecule encounters its complementary reporter in the cytosol [110]. However, recent applications also show that the readout depends on how the reporter cargo is designed. For example, split luciferase endosomal escape quantification-based analysis of pH-responsive nanoparticles showed different escape profiles when peptide tag was released as a small reporter cargo or remained linked to a larger carrier [111]. Thus, split-reporter assays provide strong evidence when the reporter design matches the therapeutic question.

When applied in fixed-cell or live-cell fluorescence microscopy, these probes enable spatiotemporal tracking of escape events, allowing investigators to correlate the timing and frequency of endosomal disruption with specific engineering features of the delivery vector. Fluorescence-based assays should include dye-only controls, quenched and unquenched calibration controls, free-cargo controls, leakage controls, lysosomal pH controls, and orthogonal validation with nonfluorescent functional readouts [112]. Without these controls, diffuse fluorescence or reduced colocalization with lysosomes should not be interpreted as efficient endosomal escape.

#### Functional readouts for cytosolic delivery

Functional readouts are essential because they test whether delivered cargo produces the expected biological effect after uptake. They are most informative when matched to the intended action of the delivered cargo. Recent engineered exosome studies illustrate this point. Liang et al. [113] evaluated intracellular delivery using Cre-reporter recombination, Cas9/single guide RNA genome editing, protein detection, dose-response analysis, and in vivo reporter activation, while Wang et al. [114] assessed hybrid exosomes-mediated mRNA delivery by linking mRNA loading with functional protein expression and endosomal escape analysis. These examples suggest that higher-tier assays should be selected according to the cargo and biological question rather than treated as a fixed checklist. Gene-regulatory cargos are better supported by target modulation or editing outcomes, protein or enzyme cargos by activity restoration, and in vivo studies by connecting tissue exposure with cargo-dependent pharmacodynamic effects.

**Fig. 6.**
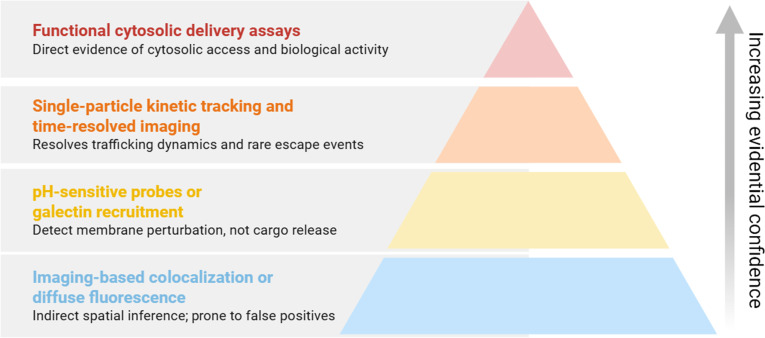
Evidence hierarchy for endosomal escape. The pyramid diagram outlines the common detection indicators used to infer endosomal escape, arranged in order of increasing strength of evidence. The lower levels rely on signals based on indirect imaging, the middle levels report endosomal membrane perturbations or vesicle dynamics, and the top level emphasizes functional tests that demonstrate entry into the cytoplasm. However, even the top-tier functional assays require cross-validation. This pyramid should be read as a guide to relative evidentiary strength. The most informative assays are those that connect the expected cargo activity with appropriate localization or escape evidence, rather than those that simply occupy the top tier.

In addition to nucleic acid reporters, enzymatic activity rescue assays can be used to assess functional cargo delivery. In this approach, exosomes loaded with an active enzyme are introduced into cells deficient in that specific enzyme. Restoration of enzymatic activity indicates that the cargo has reached a functionally accessible compartment and retained activity, although the relevant compartment may be cytosolic, lysosomal, or organelle-specific depending on the enzyme and disease model [115,116]. Enzyme rescue assays also require controls for extracellular enzyme activity, surface-bound enzyme, uptake-independent substrate conversion, and differences in enzyme stability [117]. Therefore, they are strong functional assays but not always direct cytosolic release assays. Together, these functional assays complement imaging-based methods (Fig. 6).

Recent nonexosomal nanoplatforms also provide useful methodological references for evaluating cytosolic delivery. For example, Wang et al. [118] developed tea polyphenol nanoparticles based on epigallocatechin gallate self-polymerization for cytosolic protein delivery. This system protected urate oxidase from enzymatic degradation, preserved its native structure and activity, and enabled glutathione-triggered cytosolic release, leading to therapeutic effects in hyperuricemia and gouty arthritis models. Such evidence illustrates the value of combining cargo protection, stimuli-responsive release, enzyme activity preservation, and disease-relevant functional outcomes when assessing intracellular protein delivery.

Similarly, size-selected DNA hydrogel-based systems have been developed for intracellular delivery and mRNA imaging. Although this strategy is primarily a nucleic acid-sensing platform rather than a protein therapeutic carrier, it highlights the importance of intracellular accessibility, probe stability, and functional signal generation in living cells [119]. These examples are not exosome-based, but they all suggest that engineered exosome studies should not rely solely on uptake or colocalization but should incorporate cargo-specific functional readouts such as enzymatic activity, reporter activation, target mRNA or protein modulation, and live-cell molecular sensing.

### Potential Golgi-associated sorting, recycling, and resecretion

Most engineered exosome studies interpret postuptake fate within the endosomal–lysosomal axis, with particular attention to lysosomal degradation or endosomal escape. Golgi-associated retrograde transport and recycling pathways are well established for cellular cargos, toxins, receptors, and membrane proteins, but their contribution to engineered exosome delivery remains insufficiently demonstrated. Therefore, Golgi-associated transport should currently be considered an emerging and context-dependent possibility rather than a confirmed route of engineered exosome delivery.

#### Limited evidence and interpretive boundaries

Evidence from general membrane trafficking biology suggests that selected receptors, toxins, lipids, and membrane proteins are retrieved from endosomes to the trans-Golgi network (TGN) through retromer, sorting nexins, Rab GTPases, and related tethering machinery [96,120–127]. These studies provide a conceptual basis for considering nonlysosomal sorting after exosome uptake. However, they do not suggest that engineered exosomes or their therapeutic cargos will routinely enter the TGN pathway.

At present, strictly validated examples of engineered exosomes intentionally using TGN retrograde transport to improve therapeutic delivery are lacking. The existing evidence more often supports recycling, resecretion, or redistribution of EV-associated components rather than a clearly defined Golgi-dependent functional delivery route [128]. Therefore, Golgi-associated signals should be interpreted cautiously unless they are linked to pathway perturbation and cargo-specific functional outcomes.

#### Experimental strategies and practical constraints

If Golgi-associated sorting or resecretion is proposed in engineered exosome studies, the experimental goal should be able to distinguish among 3 outcomes, including lysosomal degradation, effective cargo release, and the recovery or resecretion of the exosome source components. Pulse-chase assays combined with total internal reflection fluorescence or spinning-disk live-cell imaging have been used to resolve fast recycling routes in general endocytic trafficking [129,130]. In engineered exosome studies, similar approaches could help test whether internalized exosomes components reappear at the plasma membrane or are resecreted but requires careful separation of recycled signal from residual surface-bound exosomes.

In addition to fluorescence readouts, biochemical surface biotinylation assays provide a robust method for tracking cargo cycling. In a typical protocol, cell surface proteins are initially labeled with a membrane-impermeant and cleavable biotinylation reagent. After allowing time for endocytosis and potential recycling in a label-free medium, a membrane-impermeant reducing agent is applied to strip the biotin tag only from proteins that remained on the surface. Therefore, biotinylated proteins that are internalized and subsequently recycled to the cell surface can be specifically isolated and quantified via streptavidin pulldown followed by Western blot analysis [131]. Differences between internalization and surface reappearance can suggest recycling, but they do not by themselves distinguish recycling endosomes from Golgi-associated routes. Additional perturbation of retromer, sorting nexin, Rab11, Rab7, or TGN-associated machinery is required before assigning the route [121,122].

Emerging approaches also use advanced taggable cargo reporters [128] that undergo posttranslational modification only following resecretion, such as reporters fused to split luciferase fragments or protease-activatable fluorescent reporters, enabling sensitive detection of cargo exposure to the extracellular environment. Super-resolution organelle probes may help visualize spatial relationships between exosome-derived signals and TGN-associated compartments [132]. However, these approaches are technically demanding, require specialized instrumentation and quantitative image analysis, and remain insufficient without functional or perturbation-based validation.

Overall, Golgi-associated sorting should be presented as a potential postuptake fate that may compete with degradation or productive cargo release, not as an established route of engineered exosome delivery.

## Methodological Standards and Common Pitfalls

The methodological challenge in engineered exosome research is not merely the absence of assays, but the frequent mismatch between the evidence generated by an assay and the conclusion drawn from it [50]. For example, fluorescence colocalization may support intracellular association but does not prove cargo release. Increased tissue accumulation may indicate altered biodistribution but not functional delivery. Therapeutic improvement may reflect free cargo contamination, altered pharmacokinetics, or indirect immunomodulation rather than productive intracellular transfer. Therefore, the central task is to match each claim with the appropriate level of evidence.

### Methodological consistency under engineering constraints

Methodological standardization is a prerequisite for reproducible evaluation and translational interpretation of engineered exosome platforms [23].

In the study of genetically engineered exosomes, the genetic manipulation itself constitutes the primary experimental variable. The construction of genetically engineered exosomes typically relies on fusion proteins, overexpression systems, or artificial loading strategies, which may alter the composition, size distribution, and surface characteristics of the exosomes. Therefore, merely adopting the characterization and evaluation protocols for natural EVs is often insufficient to support conclusions about functional enhancement. Methodological consistency is therefore essential to ensure that observed effects arise from the intended engineering strategy rather than secondary consequences of construct expression [23]. Clear separation between successful construct incorporation and preservation of native exosome-associated behavior is required, as changes in vesicle abundance, labeling efficiency, or protein loading can otherwise be misinterpreted as functional improvement. It is essential to clearly distinguish between 2 distinct levels: “whether the engineered construct is successfully expressed” and “whether the engineered exosome maintains the expected biological behavior”. These must be verified separately in experimental design. Without this distinction, it is easy to mistake the effects of construct expression itself for alterations in the exosome delivery mechanism.

Quantitative normalization and instrument calibration further underpin reproducibility and cross-study comparability [133]. Techniques commonly used in engineered exosomes research, including nanoparticle tracking analysis and flow cytometry, are highly sensitive to acquisition settings and data processing choices [50,134]. Consistent calibration and particle-normalized reporting are particularly important in engineered systems, where even modest shifts in vesicle properties can disproportionately influence quantitative readouts.

### Labeling and tracing artifacts exacerbated by engineering strategies

Fluorescent labeling and reporter gene tracking technologies are widely used to monitor the uptake and intracellular fate of engineered exosomes [90,135,136]. However, these methods are highly prone to artifacts when combined with genetic engineering [137]. Lipophilic dyes may undergo nonspecific interactions with engineered membrane properties [138], while fusion fluorescent proteins themselves can alter membrane protein topology or exosome sorting efficiency [139,140]. Bright endosomal puncta frequently dominate fluorescence images, necessitating careful controls and complementary quantification strategies [141]. Without appropriate engineered negative controls, data from uptake and localization experiments are highly susceptible to misleading false-positive signals [135]. To mitigate these artifacts, researchers should incorporate complementary labeling strategies, validate that fusion tags do not alter functional properties, and include appropriate negative controls such as unlabeled or minimally tagged constructs. Such rigorous evaluation enhances confidence that observed trafficking patterns reflect biological processes rather than labeling-induced perturbations.

### Interference of engineered constructs with intracellular trafficking readouts

Engineered exosomes are often designed to enhance endocytosis, promote cytoplasmic delivery, or achieve organ-specific targeting. However, these functional improvements are frequently difficult to confirm solely through colocalization or imaging results. Overexpression of fusion tags itself may cause abnormal endosomal retention or divert vesicles toward nonphysiological transport pathways, thereby obscuring assessments of delivery efficiency. Consequently, in engineered systems, endosomal colocalization or signal diffusion alone is insufficient as evidence of functional delivery and must be cross-validated with functional readouts.

### Distinguishing functional cytosolic delivery from false-positive signals

Cytoplasmic delivery assessment represents one of the most technically challenging aspects of engineered exosome research. When evaluating whether true cytoplasmic delivery has been achieved, the risk of false positives is particularly prominent. Altered endosomal membrane permeability, dye leakage, or fusion protein release may all generate signals mimicking “cytoplasmic distribution” [110,136]. Consequently, there is a growing emphasis within the field on employing orthogonal reporting systems and highly sensitive detection methods [132], such as split-reporter complementation systems [111,142], enzymatic activity restoration, or transcription- and translation-dependent readouts. These approaches facilitate distinguishing between “signal visibility” and “functional accessibility”, thereby preventing overinterpretation of engineering outcomes.

### Context dependence and in vitro–in vivo discrepancies

Engineering-dependent advantages observed in simplified in vitro systems frequently fail to translate directly to in vivo contexts. Factors such as altered ligand exposure, construct stability, immune recognition, and clearance mechanisms can reshape the behavior of engineered exosomes following systemic administration. Accordingly, conclusions regarding targeting specificity or delivery efficiency should be interpreted within the biological context in which they were obtained [90,143], and claims of engineering benefit should be supported by validation across complementary model systems whenever possible.

### From construct validation to functional confirmation

The core objective of engineered exosomes research lies not in demonstrating the existence of engineered constructs but in proving their functional delivery under physiologically relevant conditions. Methodologically, this requires a stepwise validation strategy, including particle-normalized uptake analysis, pathway-specific perturbation, intracellular fate mapping, cargo-activity measurement, and disease-relevant functional rescue. Integrating rigorous controls, orthogonal validation strategies, and function-centered readouts substantially reduces the risk of overinterpreting engineering effects and enhances the reliability and translational relevance of methodologies based on engineered exosomes.

## Biomedical Applications of Engineered Exosome and Delivery Evidence

Currently, there have been numerous studies on the application of engineered exosomes in the biomedical field. This section does not aim to provide a catalogue of engineered exosome therapies. Instead, the selected examples are used to examine how different application studies support their delivery claims. In this context, therapeutic efficacy alone is not treated as proof of functional delivery, because improved outcomes may also arise from altered pharmacokinetics, passive tissue accumulation, residual free cargo, or indirect immunomodulation. We therefore assess the strength of evidence according to whether a study connects 3 related but nonequivalent levels: target exposure or cell association, intracellular access of the vesicle or cargo, and cargo-dependent biological activity. Evidence is stronger when these levels are linked within the same experimental system, rather than inferred from uptake or biodistribution alone.

### Applications in cancer-targeted therapy

Cancer therapy is often limited by poor drug specificity, systemic toxicity, and the immunosuppressive tumor microenvironment. Engineered exosomes have been widely explored as delivery vehicles for chemotherapeutic drugs, nucleic acids, and immunomodulators due to their unique advantages. Their membrane composition and engineering flexibility allow tumor-oriented accumulation, receptor-associated uptake, and modulation of local immune responses.

For small-molecule chemotherapy, several studies have provided useful evidence at the levels of tumor exposure and therapeutic response. AA-PEG-modified exosomes loaded with PTX and iRGD-Lamp2b exosomes loaded with doxorubicin showed improved tumor association, altered biodistribution, and enhanced antitumor efficacy [38,144]. These findings support the value of surface engineering for tumor-targeted drug delivery. Their interpretation could be further strengthened by linking tumor accumulation with drug activity in the intended cell population, such as measuring drug levels in sorted tumor cells or tumor-associated stromal cells together with pharmacodynamic markers of PTX or doxorubicin activity.

Nucleic acid delivery in cancer therapy requires more cargo-specific evidence because the therapeutic claim depends on sequence-specific molecular activity. Apo-A1-engineered exosomes delivering miR-26a to HepG2 cells were supported by increased miR-26a levels and downstream cell cycle effects, providing a clear in vitro example of cargo-related function [29]. In vivo studies of engineered exosome-mediated Kras^G12D^ siRNA delivery and CRISPR/Cas9 delivery further linked gene knockdown or editing-related outcomes with antitumor activity [145,146]. ExoASO-STAT6 provides another strong example, where antisense oligonucleotide (ASO) delivery was associated with signal transducer and activator of transcription 6 (STAT6) pathway modulation, macrophage reprogramming, and antitumor immunity in vivo [147]. For these studies, the most useful additional evidence is not simply more uptake imaging, but target engagement in defined recipient cells, such as target gene changes in tumor cells or tumor-associated macrophages, and rescue or pathway reversal experiments when feasible.

**Fig. 7.**
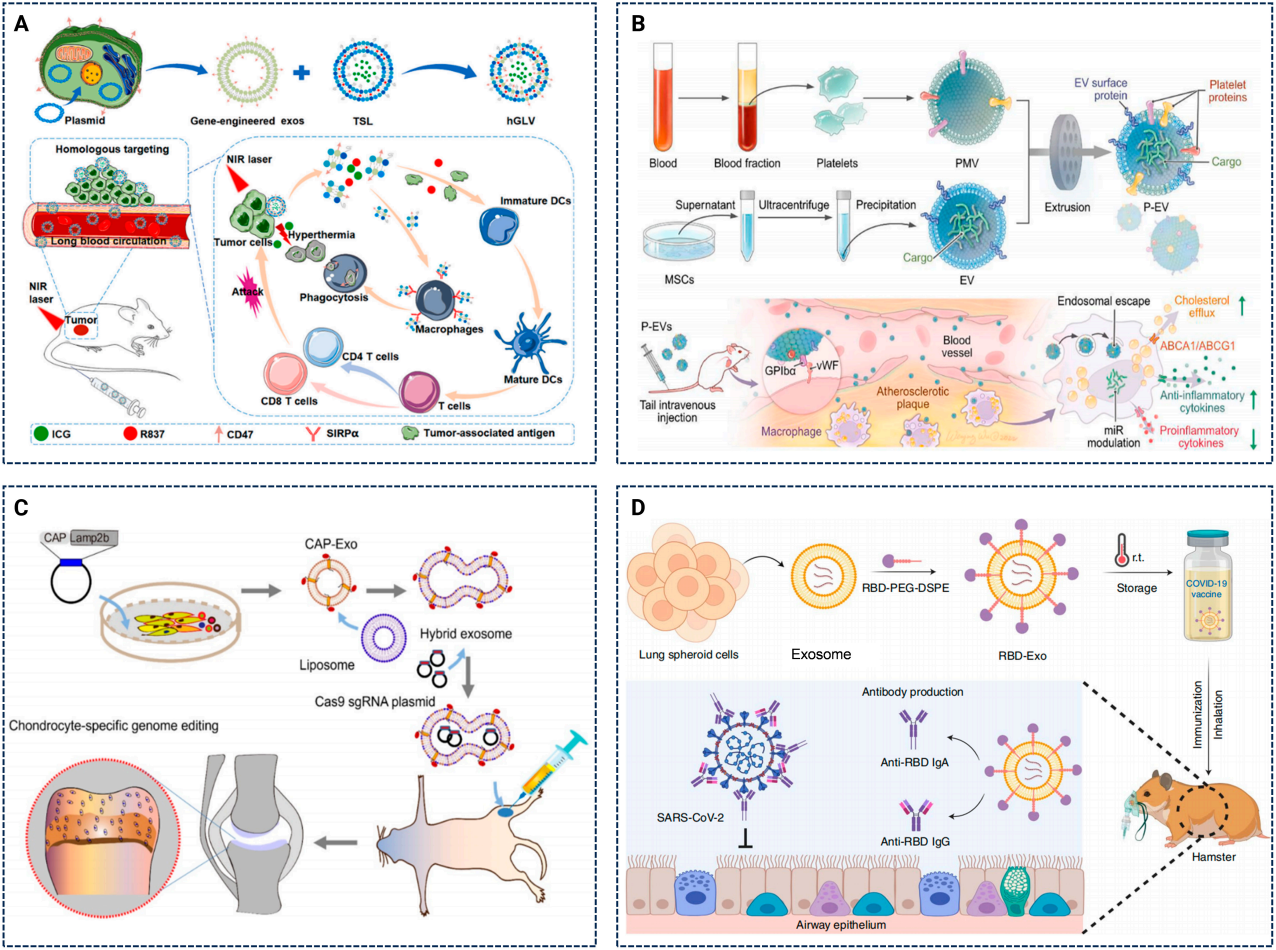
Biomedical applications of engineered exosomes. (A) Genetically engineered exosome-thermosensitive liposome hybrid nanovesicles generate local high temperature and reactive oxygen species (ROS) under near-infrared laser irradiation, triggering the apoptosis of tumor cells. Reproduced from [150] with permission. © 2021 Elsevier Ltd. All rights reserved. (B) Targeted delivery of engineered exosomes to plaques for cardiovascular disease treatment. Reproduced from [156] with permission. © 2022 Published by Elsevier B.V. (C) Chondrocyte-specific genome editing by hybrid exosomes. Reproduced from [161] under CC BY 4.0. © 2022 The Authors. (D) Fabrication of a receptor-binding domain-decorated exosome (RBD–Exo) vaccine for pulmonary delivery by inhalation. RBD-Exo induces mucosal immunity and systemic immunity with the generation of RBD-specific immunoglobulin A (IgA) and IgG responses against severe acute respiratory syndrome coronavirus 2 (SARS-CoV-2) infection in hamsters. Reproduced from [169] with permission. © 2022 The Author(s), under exclusive license to Springer Nature Limited. TSL, thermosensitive liposome; hGLV, gene-engineered exosome–thermosensitive liposome hybrid nanovesicle; RBD, receptor-binding domain; sgRNA, single guide RNA.

Engineered exosomes can also act through extracellular, membrane-proximal, or immune-mediated mechanisms, where cytosolic cargo release is not always the central question. synthetic multivalent antibody retargeted exosomes bridge CD3-positive T cells and human epidermal growth factor receptor 2-positive tumor cells to induce antitumor immunity [148]. Exosomes modified with superparamagnetic iron oxide nanoparticles use magnetic enrichment together with membrane tumor necrosis factor-alpha/tumor necrosis factor receptor signaling [149]. Hybrid exosome–liposome systems loaded with photothermal agents provide spatiotemporal control of local release and immunogenic tumor killing [150] (Fig. 7A). Engineered exosome-like nanovesicles have also been used to reshape the tumor microenvironment and promote tumor ferroptosis [151]. For these platforms, evaluation should match the intended mode of action. T-cell engagement, cytokine production, immune-cell infiltration, magnetic localization, photothermal conversion, or ferroptosis-related markers may be more relevant than assays designed only for cytosolic escape. This point is consistent with the methodological framework in the previous section. The strongest evidence is not a fixed set of experiments but evidence that matches engineered functions and therapeutic mechanisms.

### Applications in cardiovascular and cerebrovascular diseases

In cerebrovascular disease, injured myocardium, ischemic brain tissue, atherosclerotic plaques, and hypertensive pulmonary arteries often create local signals that favor vesicle accumulation. Such accumulation is valuable for therapy and when connected with the intended recipient cell type and the expected cargo activity, its interpretation becomes clearer.

In myocardial infarction and ischemia/reperfusion (I/R) injury, engineered exosomes have mainly been designed to improve cardiac homing and local repair. Wang et al. [152] genetically engineered MSC-derived exosomes to express an ischemic myocardial targeting peptide (CSTSMLKAC) fused with Lamp2b, which increased accumulation in ischemic myocardium and improved cardiac function after myocardial infarction. This study supports the value of peptide-guided cardiac targeting. If future therapeutic cargos are incorporated into a similar platform, cell-type-resolved analysis in cardiomyocytes, endothelial cells, or cardiac fibroblasts could further clarify which recipient population contributes most to functional recovery. Similarly, Gu et al. [153] used cardiomyocyte-specific peptide-modified exosomes to deliver miR-302 in an I/R model, linking cardiomyocyte-targeted uptake with Hippo/Yap pathway regulation and improved cardiac outcomes. In this case, the delivery interpretation is stronger because the study connects targeting, cargo-related signaling, and functional rescue. Further validation through miR-302 target engagement in isolated cardiomyocytes, or pathway rescue experiments, would make the causal link between exosome delivery and Hippo/Yap-mediated repair even clearer. Earlier work using cardiac homing peptide-expressing exosomes mainly established cardiac tropism [154]. This is useful for platform development, and its translational value would be extended by pairing the homing strategy with a defined therapeutic cargo and cargo-specific activity readouts.

For cerebrovascular diseases, BBB remains a major barrier for delivering small molecules and biologics into ischemic brain tissue. Tian et al. [10] reported that c(RGDyK)-functionalized exosomes could deliver curcumin to ischemic brain regions by targeting integrin αvβ3, which is highly expressed on ischemic cerebral vascular endothelial cells. This study combined lesion accumulation with anti-inflammatory and antiapoptotic outcomes, supporting the therapeutic value of engineered exosomes. The mechanistic interpretation of curcumin-related neuroprotection could be further enriched by measuring drug exposure and response in defined brain cell populations, such as endothelial cells, neurons, microglia, or infiltrating immune cells. In addition, recent work by Lan et al. [155] showed that curcumin-pretreated olfactory mucosa-derived MSCs mitigated cerebral I/R injury-induced neuronal PANoptosis by modulating microglial polarization, which provides useful mechanistic context for curcumin-based neuroprotection in ischemic brain injury. This type of complementary evidence can help connect exosome-mediated brain delivery with disease-relevant cellular pathways.

Engineered exosome-based systems have also been explored in chronic vascular diseases. Platelet membrane-modified EVs showed plaque homing, macrophage uptake, and plaque regression in atherosclerosis models [156] (Fig. 7B). In this setting, macrophage uptake is highly relevant because plaque macrophages are central drivers of inflammation and lipid handling. The interpretation could be strengthened by linking vesicle uptake in plaque macrophage subsets with changes in inflammatory phenotype, cholesterol excretion, or clearance of apoptotic cells. Cabbage exosome-like nanoparticles encapsulating transfer RNA-derived small RNA were also reported to prevent postinjury arterial restenosis [157], suggesting that plant-derived exosome-like carriers may have value in vascular repair. For this type of platform, local retention and target-cell response in vascular smooth muscle cells or endothelial cells would be especially informative. In pulmonary arterial hypertension, CAR peptide (CARSKNKDC)-modified EVs targeted hypertensive pulmonary arteries and inhibited abnormal smooth muscle cell proliferation [158]. Because smooth muscle remodeling is a key feature of pulmonary arterial hypertension, this study would be further supported by connecting pulmonary artery targeting with cargo-dependent signaling changes in pulmonary arterial smooth muscle cells.

Overall, cardiovascular and cerebrovascular applications show that the useful evidence is not always the same across disease models. For cardiac repair, recipient-cell identity and pathway rescue are especially important. For ischemic brain injury, brain-region exposure and cell-type-specific neurovascular responses help clarify therapeutic mechanisms. For chronic vascular disease, plaque macrophage function, vascular smooth muscle remodeling, or endothelial repair may be more relevant than a generic uptake assay. This disease-specific interpretation connects the application studies with the methodological framework discussed above while respecting the therapeutic goals of each study.

### Applications in inflammation

Inflammation is a common core pathological process shared by a variety of acute and chronic diseases, but the relevant target cells and therapeutic mechanisms differ widely across disease settings. Native exosomes already have immunomodulatory activity, while engineered exosomes can further improve local retention, immune-cell targeting, or the display and delivery of anti-inflammatory molecules [159]. In this section, the key question is therefore whether the assays used are well matched to the intended anti-inflammatory mechanism.

In autoimmune and degenerative inflammatory diseases, engineered exosomes have been used to regulate macrophages, chondrocytes, and local tissue responses. For rheumatoid arthritis, You et al. [160] used metabolically engineered stem cell-derived exosomes to modulate macrophage heterogeneity and promote a shift from proinflammatory M1 phenotype to an alternatively activated macrophage (M2) phenotype, thereby reducing local inflammation. This study is well aligned with an immune-reprogramming mechanism. Its interpretation could be further strengthened by connecting exosome uptake in macrophage subsets with cytokine changes and macrophage-state markers within the same joint microenvironment. In osteoarthritis, chondrocyte-affinity peptide-engineered hybrid exosomes delivering CRISPR/Cas9 to chondrocytes linked targeting with matrix metalloproteinase-13-related genome editing and phenotypic rescue [161] (Fig. 7C). For this type of gene-editing platform, the most useful additional evidence would be cell-type-resolved editing efficiency in joint tissue, together with matrix-protection markers in the edited chondrocyte population. Another osteoarthritis study showed that human amniotic epithelial stem cell-derived exosomes deliver ACTA2 antisense RNA 1 (ACTA2-AS1) to chondrocytes and suppress ferroptosis by targeting ACSL4 (acyl-CoA synthetase long-chain family member 4) [162]. Further rescue experiments, such as restoring ACSL4 signaling or modulating ferroptosis in recipient chondrocytes, would help clarify how much of the therapeutic effect depends on this RNA-mediated pathway.

Engineered exosomes are also being developed for systemic inflammatory diseases. In cytokine storm-driven disorders such as sepsis, Gupta et al. [163] displayed decoy receptors for tumor necrosis factor-alpha and interleukin-6 on exosome surfaces, allowing the vesicles to neutralize proinflammatory cytokines and reduce systemic inflammation. For this type of surface-function platform, cytosolic release is not the main question. More relevant validation includes receptor display density, cytokine-binding capacity, circulation stability, and suppression of inflammatory cytokine signaling in disease models. Choi et al. [164] used exosome-mediated delivery of super-repressor inhibitor of nuclear factor kappa B alpha to reduce sepsis-associated organ injury and mortality. Because this strategy acts on the nuclear factor kappa B pathway, its interpretation would be especially clear when nuclear factor kappa B alpha delivery is linked with reduced nuclear nuclear factor kappa B activity in defined recipient cells and with organ-level inflammatory injury markers.

Overall, the application of engineered exosomes in inflammatory diseases has expanded from the simple delivery of anti-inflammatory molecules to multiple levels, including immune cell reprogramming, regulation of the inflammatory microenvironment, and promotion of tissue repair. Exosome engineering not only enhances the targeting and efficacy of anti-inflammatory therapies but also provides a new strategic foundation for precision interventions in complex inflammatory diseases.

### Other biomedical applications of engineered exosomes

In addition to cancer, cardiovascular, and inflammatory diseases, engineered exosomes have also been explored for neurological disorders, tissue repair, enzyme replacement, and vaccination. These applications differ greatly in their therapeutic goals, so the most useful validation strategy also differs. For some systems, the key question is whether vesicles cross a biological barrier and reach the right tissue. For others, it is whether a protein, enzyme, RNA, or surface antigen remains active in the relevant biological setting.

In the treatment of CNS disorders, engineered exosomes are often designed to cross the BBB or improve delivery to selected neural cell populations. Alvarez-Erviti et al. [9] used RVG-Lamp2b exosomes to deliver BACE1 siRNA to the mouse brain, with reduced BACE1 mRNA and protein providing cargo-related evidence beyond brain accumulation alone. More recent RVG-brain-derived neurotrophic factor-exosomes further extend this idea by linking neural targeting with neurogenesis-related outcomes in depression models [165]. For CNS applications, future studies would be strengthened by identifying which brain cell populations receive the engineered vesicles and by connecting this with cell-type-specific target modulation or neurofunctional outcomes. The EXOtic (exosomal transfer into cells) system is another useful example, as producer-cell engineering, catalase mRNA and protein delivery, and neuroprotective effects were evaluated together in a Parkinson’s disease model [166]. In such systems, the most informative follow-up would be to connect catalase activity in relevant neural cells with oxidative stress reduction and behavioral benefit.

In tissue regeneration, engineered exosomes are increasingly combined with biomaterials to improve local retention and reshape the microenvironment. Hydrogel-encapsulated engineered exosomes have been used to support endoplasmic reticulum homeostasis and promote diabetic bone regeneration, while hypoxia-challenged sEV-engineered nanofiber scaffolds accelerated diabetic wound healing by improving the function of skin repair cells [167,168].

Engineered exosomes also have promise in inherited metabolic disorders, where enzyme activity offers a relatively direct functional readout. Taking lysosomal storage diseases as an example, EVs derived from recombinant cell factories have been used to deliver deficient enzymes, including alpha-galactosidase A and N-sulfoglucosamine sulfohydrolase, and reduce substrate accumulation in tissues such as the brain and kidneys, that are difficult to fully target with conventional enzyme replacement therapy [115]. This type of study is particularly informative because enzyme activity and substrate reduction are closely linked to the expected therapeutic mechanism. Further enzyme activity analysis resolved tissue or cell type would help clarify where the delivered enzyme is most active and which organs benefit most from exosomes-mediated distribution.

Furthermore, engineered exosomes are also being developed as vaccine platforms. Wang et al. [169] decorated lung-derived exosomes with the severe acute respiratory syndrome coronavirus 2 receptor-binding domain, constructing an inhalable vaccine that enhanced antigen retention in the respiratory tract and induced both mucosal and systemic immune responses (Fig. 7D). For this type of surface-antigen platform, more relevant evidence includes antigen stability on vesicles, uptake by antigen-presenting cells, mucosal immunoglobulin A responses, systemic immunoglobulin G responses, T-cell activation, and protection after viral challenge. This example shows that engineered exosomes can act not only as intracellular delivery vehicles but also as extracellular antigen display and immune activation platforms.

Overall, the applications of engineered exosomes span multiple fields. With the continuous refinement of delivery strategies, payload design, and quality control systems, engineered exosomes are expected to develop further as a next-generation drug delivery platform. At the same time, their evaluation also needs to evolve with the complexity of these applications. Methodological strategies should be selected according to the therapeutic goal, cargo type, and expected site of action, so that engineering design, delivery behavior, and biological effect can be interpreted within the same experimental context.

## Conclusion and Future Perspectives

Engineered exosomes have moved from simple carrier design toward more complex systems that combine surface engineering, cargo loading, hybrid assembly, biomaterial support, and disease-specific functions. This development has also changed their evaluation methods. A useful evaluation strategy should focus on whether the selected methods match the engineered functions, the cargo type, the expected site of action, and the therapeutic goals.

**Fig. 8.**
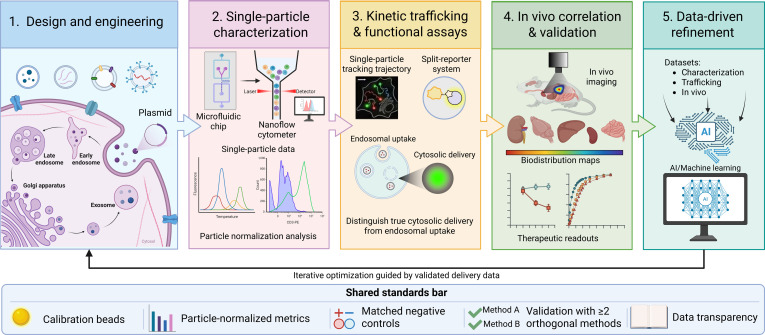
Integrated methodological pipeline for evaluation and translation of engineered exosomes. The pipeline links engineering design, vesicle characterization, intracellular trafficking analysis, functional assays, in vivo validation, and data-driven refinement. Rather than prescribing a fixed checklist, it highlights that evidence should be selected based on the type of goods, the disease model, and the expected biological effects at the appropriate stage. AI, artificial intelligence.

This review emphasizes a staged but flexible way to evaluate engineered exosome delivery. Targeting assays help define tissue exposure or cell association. Endocytic and trafficking analyses clarify how vesicles enter and move within recipient cells. Cargo-specific readouts indicate whether the delivered molecule produces the expected biological effect. The strength of evidence increases when these layers are connected within the same experimental system. In disease applications, this means that chemotherapy, RNA therapy, immunomodulation, enzyme replacement, tissue repair, and vaccine platforms may require different but equally reasonable validation strategies (Fig. 8).

As engineered exosome platforms become more heterogeneous, methods that resolve vesicle subsets become increasingly useful. Nanoflow cytometry, high-resolution nanoparticle tracking, and microfluidic single-exosome platforms can help relate ligand density, cargo incorporation, and vesicle composition to downstream cell behavior [170]. These tools are particularly suitable for addressing the overall measurement masking of subgroup or batch differences.

Data-driven approaches may further support this process, but they must be based on the actual situation. Rather than replacing experimental validation, artificial intelligence-assisted strategies such as machine learning models could help organize variables such as donor-cell source, vesicle surface markers, ligand density, cargo loading, target cell receptor profiles, trafficking patterns, and functional readouts [171,172]. Predictions from such models should be treated as hypotheses to be tested with well-matched experimental assays.

Overall, the next step for engineered exosome research is not only to build more complex delivery systems but also to evaluate them in a way that fits their application goals. When engineering design, delivery behavior, and biological effect are interpreted together, engineered exosomes can be developed with clearer mechanisms, stronger reproducibility, and a more realistic path toward translation.

## Data Availability

Not applicable.
